# Characterization of military police officers of Alagoas affected by COVID-19

**DOI:** 10.47626/1679-4435-2022-778

**Published:** 2022-03-30

**Authors:** Vanessa Lôbo de Carvalho, Deivson Cavalcante Gomes de Oliveira, Marcelo Oliveira Silva, Joana Darc Gomes de Oliveira, Leandro Eugênio Ferreira da Silva

**Affiliations:** 1 Curso de Fisioterapia, Universidade Estadual de Ciências da Saúde de Alagoas, Maceió, AL, Brazil.; 2 Diretoria de Saúde, Polícia Militar do Estado de Alagoas, Maceió, AL, Brazil.; 3 Coordenação de Qualidade de Vida no Trabalho, Universidade Federal de Alagoas, Maceió, AL, Brazil.

**Keywords:** COVID-19, occupational health, police, epidemiological monitoring

## Abstract

**Introduction:**

COVID-19 has greatly affected society by limiting the functioning of sectors of the economy and public services. Considering the essential character of many of these services, especially public security, it is necessary to understand how the disease has affected different groups within the population so that public policies for facing this problem can be implemented.

**Objectives:**

To identify and describe the profile of military police officers affected by COVID-19.

**Methods:**

This is a descriptive observational study with a quantitative approach, based on secondary data. The electronic medical records of 737 military police officers affected by COVID-19 were accessed; sociodemographic, biological, and professional data were collected, as well as data on disease progression. Data were analyzed using Bioestat^®^ software, v5.3.

**Results:**

The peak of the COVID-19 contagion curve happened first among military police officers of the state of Alagoas than in the general population, and a positive effect of social distancing was observed in the containment of disease spread. Moreover, specialized operations units had a higher contagion rate in view of the higher level of exposure linked to their work activities.

**Conclusions:**

This study described the profile of military police officers affected by COVID-19, which can substantiate the adoption of public policies and new strategies to fight this disease among officers in Alagoas, thus ensuring the continuity of the service provided to society.

## Introduction

According to the Brazilian Federal Constitution of 1988,^1^ the military police is responsible for overt policing and maintaining public order. Overt policing is related to the uniform presence in society in order to prevent crime and arrest criminals. By acting in the preservation of public order, police activity seeks to ensure citizens their fundamental rights and guarantees, the functioning of public institutions, and public and private property.

The military police activity, by itself, presents risks to the quality of working life and health of public security professionals as they deal with violence and the risk of death daily, in addition to overwork, inadequate work conditions, and stress.^2^

In December 2019, the severe acute respiratory syndrome coronavirus 2 (SARS-CoV-2) was identified, causing COVID-19 that spread through the population and culminated in the current pandemic.^3^

COVID-19 presents a varied clinical picture and can progress asymptomatically to severe conditions requiring hospitalization and, in 5% of cases, ventilatory support.^4^ The COVID-19 pandemic has affected the whole society by limiting the functioning of sectors of the economy and public services. Moreover, given the essential nature of certain activities, in particular public security, the COVID-19 pandemic can also be considered an occupational hazard.

The working conditions of military police officers expose them to a higher chance of health problems that may lead to the onset of diabetes, hypertension, and other diseases that may worsen COVID-19. As the transmission of COVID-19 happens by contact with saliva droplets and air, the chances of transmission through sneezing and coughing are increased, making its contagion rate very high.^4^ The police officer occupation is one of the most vulnerable to contagion because these are considered frontline workers.^5^

It is noteworthy that the military police received another attribution during the pandemic context: to ensure compliance with government decrees of isolation and social distancing. These decrees were issued in March 2020 when the first case of the disease emerged in the state of Alagoas, with the aim of fighting the spread of COVID-19. The military police thus increased its activities within the scope of overt policing and guaranteeing law and order through decrees such as Decree no. 69541 of March 19, 2020. This decree declared a state of emergency in the state of Alagoas and intensified measures to fight the public health emergency of international importance resulting from COVID-19 in the state of Alagoas, also providing other measures.^6^ After the expiry of this decree, others were edited by the state government to extend and adopt contingency measures regarding COVID-19.

With the increased risk of contagion, the Public Security Department of the state of Alagoas published Ordinance no. 0349/2020, which established a rapid testing protocol to detect COVID-19 among public security professionals as a complementary measure to fight the international public health emergency resulting from COVID-19.^7^ The Military Police of the State of Alagoas (Polícia Militar do Estado de Alagoas - PMAL) created and guided the execution of the COVID-19 Standard Operating Procedure (SOP), which contained guidance on how military police officers should protect themselves from the virus on and off duty according to standards of the World Health Organization (WHO) and the Brazilian Ministry of Health.

Despite the wide implementation of control measures, the ongoing pandemic has had a devastating effect.^8^ Considering the relevance of the military police service to society and the impact of the pandemic on the physical and mental health of military police officers, it is extremely important to study the population being affected, especially considering the health of workers who were acting to ensure measures of social distancing and public order. Therefore, it is necessary to characterize the military police officers who were affected by COVID-19.

## Methods

This is a quantitative study with an observational, descriptive design, based on secondary data. Our research was performed at the Hospital Medical Center of PMAL.

The sample of this study was non-probabilistic, and participants were selected by convenience. The military police officers affected by COVID-19, when presenting to the medical board of the Hospital Medical Center to resume their occupational activities, were invited to an explanation about the objectives of this research. If the participants allowed access to their electronic medical records, they signed the informed consent form. Researchers then accessed the electronic medical records of military police officers who tested positive for COVID-19 in order to fill out a form characterizing the sample regarding sex, age, blood type, education, neighborhood of residence, neighborhoods of operation, whether the participant worked at administrative or operational sectors, whether disease worsening required hospitalization, and duration of hospitalization (days). Data were arranged in a spreadsheet and were statistically analyzed using the Bioestat^®^ v5.3 statistical package; the heterogeneity of the studied variables was verified by a Kolmogorov-Smirnov test, correlations were analyzed by the Spearman test, and comparisons were performed using the chi-squared and G-tests. For all values, an alpha value lower than or equal to 5% was adopted.

The research was approved by the Research Ethics Committee of Universidade Estadual de Ciências da Saúde de Alagoas, under CAAE no. 35855720.4.0000.5011, Opinion no. 4.280.078, in September 2020.

## Results

The sample consisted of 737 military police officers contaminated with COVID-19 from March to September 2020. Although public security professionals interact directly with the population, there are currently no studies on the risks of COVID-19 infections in their work process, which hinders the adoption of strategies to minimize virus transmission.^9^ The lack of knowledge on the epidemiological behavior of COVID-19 among public security professionals affects the implementation of appropriate public policies in a way that puts their health and that of the community at risk, considering the contact this security agent has with the society during his or her professional activity. Contagion among police officers results in a decrease in the number of workers due to absences from suspected or confirmed infection, resulting in serious losses in the service to the community.^10^

**Figure 1:**
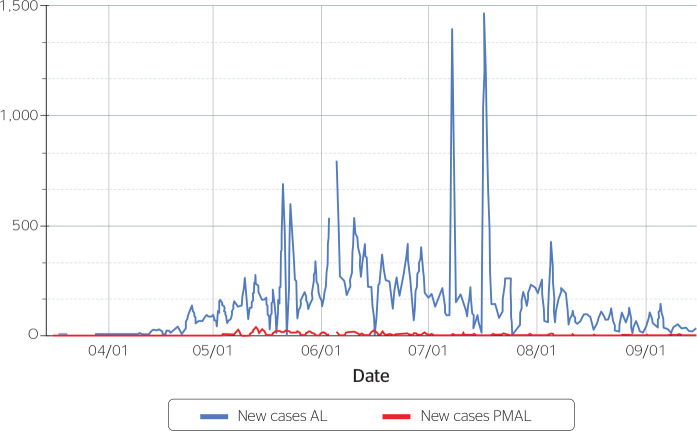
Curve of contagion/new cases among military police officers and the general population of the state of Alagoas from March to September 2020. PMAL = Polícia Militar do Estado de Alagoas.

When comparing the curve depicting contagion/daily new cases of COVID-19 among military police officers with the general society in the state of Alagoas (Figure 1), we observed that the peak of daily notifications of new cases happened first in the PMAL (May/2020) than in the general society (July/2020). This can result from the lack of social distancing imposed by their work activity. Research conducted in other countries identified that COVID-19 infections first reached their peak among police officers, which also assumed new humanitarian attributions such as collecting material for examinations and structuring logistics for moving specific resources.^5^

By analyzing the geographical distribution of military police officers contaminated by COVID-19 in the state, we observed that 507 (68%) of them lived in the state capital metropolitan area and 230 (32%) lived in the countryside. Although our sample indicated that the fraction of military police officers diagnosed with COVID-19 was higher in the capital metropolitan area, no significant difference was observed when analyzing the relationship between infected individuals and all military police officers.

The military police officers affected by COVID-19 were aged from 21 to 59 years, with a mean age of 36 ± 8.8 years. The most affected age group in both the general community and the military police force was 30 to 39 years. The higher percentage of cases among police officers in this age group can be explained by their demographic characteristics (40.69% of all officers are in this age group).

In the analyzed sample, 22 (2.98%) police officers were hospitalized, 6 (0.81%) of them in the intensive care unit (ICU). When comparing the age groups of police officers who required hospitalization, a higher incidence was observed for those aged between 40 and 49 years (Table 1). Regarding the length of hospitalization, officers remained in the hospital for a mean duration of 19 ± 24.96 days, with a maximum of 127 days and a minimum of 2 days.

**Table 1:** Comparison of confirmed COVID-19 cases by age group and hospitalization requirements of police officers from March to September 2020

Hospitalization		p-value	Age group
Yes	No		
0	129	0.0039*	18-29
22	586		Others
3	313	0.0069^†^	30-39
19	402		Others
11	172	0.0049^†^	40-49
11	543		Others
8	101	0.0091*	50-59
14	614		Others
22	715		Total

* G-test.

† Chi-squared test.

The lethality of COVID-19 in the studied period was 0.67% (5 deaths) in the PMAL and 2.4% (1991 deaths) in the general population. The lethality among police officers affected by COVID-19 was lower than that in the general population probably due to the fact that most of the infected officers were between 30 and 39 years old, a group considered to be at a lower risk of severe complications.

Out of the 737 police officers affected by COVID-19 in the investigated period, 99 (13.4%) were female and 638 (86.6%) were male; this is in agreement with the overall sex distribution in the PMAL, where 15% are female police officers and 85% are male. These data differed from the overall population of Alagoas, such as in the capital Maceió, which has 56% and 55.6% of women in the affected population and 44% and 44.4% of men in the affected population.

Regarding education, 2 (0.27%) officers had primary education, 310 (42.06%) had secondary education, 162 (21.98%) did not complete higher education, 223 (30.26%) had an undergraduate degree, 5 (0.68%) did not complete graduate studies, 32 (4.34%) had a graduate degree, and 3 (0.41%) participants did not inform their education. No differences were observed in the contaminated/total officers ratio. Lima et al.^11^ evaluated behavioral aspects and beliefs of the population before the COVID-19 pandemic in the state of Ceará and identified that people with elementary schooling complied less with social isolation measures because they considered the risk of contagion to be smaller when compared to people with higher schooling levels.

However, when comparing levels of education with hospitalization rates, that is, disease worsening, the hospitalization rate was noticeably higher among police officers with secondary education (Table 2).

**Table 2:** Confirmed COVID-19 cases by schooling level of police officers admitted from March to September 2020

Schooling	Hospitalization		p-value
	Yes	No	
Primary education	0	2	0.7275*
Others	22	713	
Secondary education	15	295	0.0117^†^
Others	7	420	
Incomplete undergraduate education	3	159	0.3115*
Others	19	556	
Complete undergraduate education	3	220	0.0849^†^
Others	19	495	
Incomplete graduate education	1	4	0.1297*
Others	21	711	
Complete graduate education	0	32	0.1590*
Others	22	683	
Did not inform	0	3	0.6695*
Others	22	712	
Total	22	715	

* G-test.

† Chi-squared test.

When considering hierarchical ranks, 72 (9.77%) were officers (colonel, lieutenant colonel, major, captain, first lieutenant, and second lieutenant) and 665 (90.23%) were non-officers (aspiring officer, sub-officer, sergeant, corporal and soldiers); 24 (3.61%) of these soldiers were students from the Soldier Training Course. The percentage of contaminated non-officers results from the greater number of individuals in this group and also the greater contact they have with society during overt policing and supervision of compliance with the social distancing enacted in the state. As a measure to mitigate COVID-19 contagion, PMAL instituted on-duty and off-duty protection guidelines according to guidelines by the WHO and the Brazilian Ministry of Health.^12^ Protocols aimed at the adequacy of police approaches were developed worldwide in order to mitigate the risk of COVID-19 contagion when approaching suspects, interrogating witnesses, and transporting prisoners.^5^

The lowest percentage of infected individuals was among students of soldier and officer training courses. These data confirm the importance of social distancing, since during data collection they were not attending in-person classes.

Regarding blood type, 372 (50.75%) of the military personnel contaminated with COVID-19 were type O, 202 (27.55%) were type A, 73 (9.95%) were type B, 61 (8.32%) were type AB, and 25 did not respond. The studied sample presented a predominance of type O blood, as well as those who required hospitalization. There was no difference in disease worsening according to the blood type of the hospitalized officers. This fact differed from a study conducted in Turkey that evaluated 397 patients admitted to the ICU considering the relationship between disease worsening and blood type; 44.3% of the patients were type A and 27.5% were type O.^13^

Regarding marital status, 300 (40.92%) of the participants were single, 309 (42.15%) were married, 1 (0.13%) was a widower, 32 (4.36%) were divorced, and 28 (3.81%) had a stable union. This information is relevant for preventive measures within the domestic context (considering that the disease has a high potential for contagion within the core family), thus avoiding dissemination at home, at work, and in the society.^6^ Among the containment measures adopted by the military police force, we note the testing of symptomatic patients and 14-day sick leaves established from the first day of symptoms, as recommended by the WHO and the Brazilian Ministry of Health.^14^

The city of Maceió has 53 neighborhoods grouped into 8 health care districts (HD) in order to optimize the management of community health care. When analyzing the neighborhoods of residence of military police officers affected by COVID-19, we observed a greater number of cases in the geographic area corresponding to the 2nd and 7th HDs.

**Figure 2:**
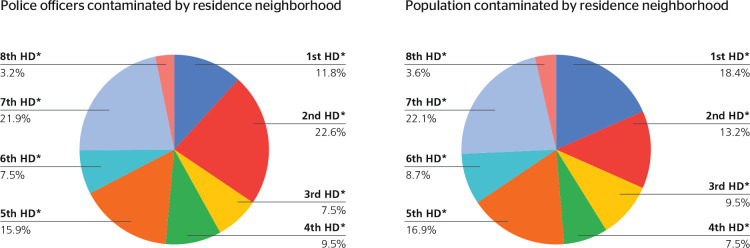
Percentage of contaminated individuals among military police officers and the general population, by health care district (HD).

When comparing the distribution of police officers contaminated by HD with the general population,^15^ we observed a higher rate of contagion in the 2nd HD and a lower rate in the 1st HD among police officers when compared to the general population. The 2nd HD comprises neighborhoods with characteristics of socioeconomic vulnerability. Therefore, we can infer that socioeconomic status may have contributed to a lower contamination rate of police officers living in these neighborhoods when compared to the general population (Figure 2). The other HD of the city of Maceió showed no differences between police officers and other inhabitants regarding contamination by COVID-19.

A higher percentage of contagion was observed among contaminated officers working in specialized operations units (14.79%), followed by the health care unit (13.14%). This situation can be explained by the fact that specialized operations officers do not have a predefined territory of operation and may act in different regions of the city and the state (Table 3). The greater contact of the police with communities at different epidemiological stages may have increased the risk of contamination by COVID-19.

**Table 3:** Comparison of contaminated individuals and total number of officers in their work units from March to September 2020

Type of unit	Contaminated	Total	% contaminated	p-value*
Operational units	427	3.793	11.26	< 0.0001
Specialized operations units	173	1.170	14.79	< 0.0001
Teaching units	51	1.081	4.72	< 0.0001
Health care unit	18	137	13.14	< 0.0001
Administrative unit	68	1.063	6.40	< 0.0001
Total	737	7.244	10.17	< 0.0001

* Chi-squared test.

Operational units, in general, and the health unit presented higher percentages of contaminated individuals than the administrative and teaching units. This may be the result of the former having direct and constant contact with society in their overt activity and, in the case of health care professionals, engaging in COVID-19 testing of public security officers. Moreover, part of the officers working at administrative units were subjected to remote work and rotating shifts, reducing the risk of contamination. As for the teaching units, in-person classes were suspended, complying with the state decree that dealt with measures of social distancing to cope with the pandemic.

Given the distribution of contaminated military police officers, it is essential to instruct that, when presenting any COVID-19 symptoms, the police officer should seek medical care and, if necessary, perform the test and remain in isolation in order to preserve his or her own life, that of his or her family, and that of the troop.^5^

## Conclusions

In light of the obtained data, we noticed that the peak of the COVID-19 contagion curve happened first among military police officers in Alagoas than in the general population due to the occupational hazard involving direct contact with society and the difficulty of performing social distancing inherent to their work activity.

Among the military police officers of Alagoas contaminated by COVID-19, there was a predominance of men aged 30 to 39 years, with secondary education, type O blood, and living in the 2nd HD, which indicated a risk of social vulnerability. Furthermore, the specialized operations units had higher rates of contagion, possibly because they did not have a predefined territory of action; this resulted in greater exposure, including in regions at different stages of contagion levels in the state.

The profile of military police officers affected by COVID-19 can substantiate the adoption of public policies and new strategies to fight the disease and protect the health of the police officers of Alagoas, ensuring the continuity of the service provided to society.
